# Immunophenotypic Lymphocyte Profiles in Human African Trypanosomiasis

**DOI:** 10.1371/journal.pone.0006184

**Published:** 2009-07-08

**Authors:** Caroline Boda, Bertrand Courtioux, Pierre Roques, Lynda Pervieux, Gédéon Vatunga, Théophile Josenando, Constant Roger Ayenengoye, Bernard Bouteille, Marie-Odile Jauberteau, Sylvie Bisser

**Affiliations:** 1 Université de Limoges, IFR 145 GEIST, Institut de Neurologie Tropicale, EA 3174 NeuroEpidémiologie Tropicale et Comparée, Faculté de Médecine, Limoges, France; 2 Centre International de Recherches Médicales de Franceville (CIRMF), BP 769, Franceville, Gabon; 3 CEA, Division of Immuno-Virology/Institute of Emerging Diseases and Innovative Therapies (iMETI), Fontenay-aux-Roses, France; 4 Université de Limoges, IFR 145 GEIST, EA 3174 NeuroEpidémiologie Tropicale et Comparée, Faculté de Médecine, Limoges, France; 5 Instituto de Combate e Controlo das Tripanossomiases-ICCT/Minsa, Luanda, Angola; 6 Programme National de Lutte contre la Trypanosomose Humaine Africaine, PNLTHA, Libreville, Gabon; 7 Université de Limoges, IFR 145 GEIST, EA 3842 Homéostasie cellulaire et pathologies, Faculté de Médecine, Limoges, France; Institut Pasteur, France

## Abstract

Human African trypanosomiasis (HAT) is a deadly vector-born disease caused by an extracellular parasite, the trypanosome. Little is known about the cellular immune responses elicited by this parasite in humans. We used multiparameter flow cytometry to characterize leukocyte immunophenotypes in the blood and cerebrospinal fluid (CSF) of 33 HAT patients and 27 healthy controls identified during a screening campaign in Angola and Gabon. We evaluated the subsets and activation markers of B and T lymphocytes. Patients had a higher percentage of CD19^+^ B lymphocytes and activated B lymphocytes in the blood than did controls, but lacked activated CD4+ T lymphocytes (CD25^+^). Patients displayed no increase in the percentage of activated CD8+ T cells (HLA-DR^+^, CD69^+^ or CD25^+^), but memory CD8 T-cell levels (CD8^+^CD45RA^−^) were significantly lower in patients than in controls, as were effector CD8 T-cell levels (CD8^+^CD45RA^+^CD62L^−^). No relationship was found between these blood immunophenotypes and disease severity (stage 1 *vs* 2). However, CD19^+^ B-cell levels in the CSF increased with disease severity. The patterns of T and B cell activation in HAT patients suggest that immunomodulatory mechanisms may operate during infection. Determinations of CD19^+^ B-cell levels in the CSF could improve disease staging.

## Introduction

Human African trypanosomiasis (HAT) is caused by *Trypanosoma brucei (T. b.) gambiense* in West and Central Africa, and by *T. b. rhodesiense* in East and Southern Africa. Epidemics of the disease occur, with the Democratic Republic of Congo, Angola and Sudan the most afflicted countries in recent years [1]. Disease control is based on the implementation of appropriate public health policies, which remains difficult in countries with limited resources subject to political unrest. Humans contract the disease after being bitten by an infected tse-tse fly. The trypanosomes move out from the site of inoculation, invading all parts of the body via the bloodstream and lymphatic system (stage 1). The encephalitic stage (stage 2) begins when parasites cross the blood-brain barrier, modifying sleep-wake cycles, endocrinological functions and behavior. Over time, the entire central nervous system (CNS) may be affected, with sensory and motor dysfunctions progressively leading to irreversible demyelinating disease [2]. It has been suggested that an autoimmune response against neuronal and myelin antigens occurs in cases of CNS involvement. Indeed, autoantibodies reacting with galactocerebrosides [3], the major components of myelin, and with neurofilaments [4], the intermediate filaments of neurons, have been reported. The time-course of the disease varies widely due to the complexities of the host-parasite interaction, resulting in immune system-driven mechanisms with both beneficial and deleterious effects on the body [5]–[6]. The immunophenotyping of lymphocytes in patients with viral infections, immunodeficiencies and autoimmune diseases has provided clues to the pathogenesis of these diseases [7]–[8]. Furthermore, the patterns of lymphocyte activation observed during the course of Chagas' disease, caused by a trypanosome endemic in South America *(Trypanosoma cruzi)*, may provide an explanation for the cardiac disease occurring during infection [9]–[10]. In HAT, the mechanisms of host-parasite interaction and the subsequent development of a demyelinating disease in the CNS are not well understood. Classically and alternatively activated macrophages play a key role in initiating the immune response and disease control [11]–[13]. Indeed, the protection of the host against lesion formation depends on the ability to downregulate alternatively activated macrophages. The subsequent stages of cellular immune profile during the course of the disease have not been extensively studied in humans and remain unclear. We therefore carried out a clinical study with the aim of describing the phenotypic changes occurring in subsets of lymphocyte during HAT.

## Results

### Enrollment of patients and clinical data

Survey 1 was performed in November 2002 in Angola (Viana and Bengo districts) and in Gabon (N'kembo Hospital). Blood samples from 17 patients (16 Angolans, 1 Gabonese) and 13 controls (Gabonese from N'kembo) were analyzed. Survey 2 was performed in June 2004 in Angola (Bengo district) and Gabon (Estuaire district). Blood samples from 16 Angolan patients and 14 controls (6 Gabonese, 8 Angolans) were analyzed. All subjects belonged to the Bantu ethnic group and were of similar socioeconomic status. They were matched for age and sex with controls whenever possible. The epidemiological and clinical characteristics and disease stages of the patients are summarized in Table 1. Briefly, clinical and biological data allowed us to identify 17 patients in stage 2 (S2), 11 patients in stage 1 (S1) and 9 patients in intermediate stage (S-int) as defined in material and methods below.

**Table 1:** Clinical and epidemiological features of the subjects studied.

Characteristics of the patients n = 33	
***Epidemiologic***	
Male	19
Female	14
Age, (mean range)	32 (12–55)
Angolan origin	32
Gabonese origin	1
Active/passive screening	10/23
***Clinical***	
Duration of signs and symptoms at enrollment	3 weeks to 3 years
Signs of hemo-lymphatic disease	18
Neurological signs	15
Sleep disturbances	11
Sensory disturbances	3
Motor disturbances	11
Psychiatric disturbances	7
***Stage determination***	
Presence of trypanosomes in CSF	17
WBC >20	13
WBC between 5–20	9
WBC <5	11

WBC = White Blood Cell Count in CSF.

### Blood lymphocyte subset profiles

#### B and T cell activation markers in blood

**Figure 1:**
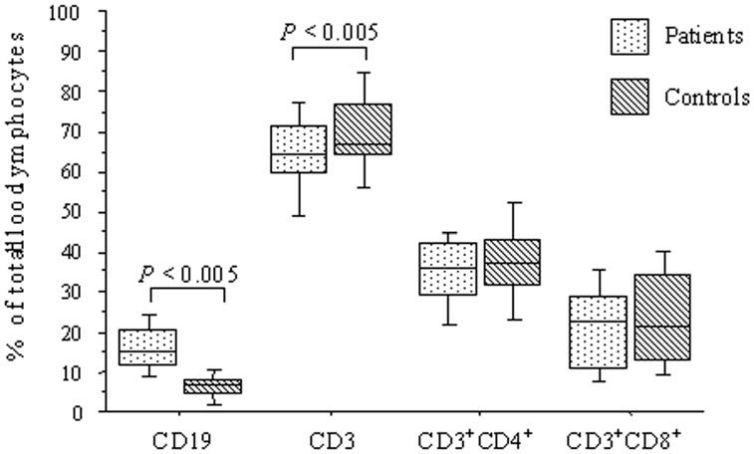
Distribution of B cells (CD19^+^), T cells (CD3^+^), and T-cell subpopulations (CD3^+^CD4^+^ or CD3^+^CD8^+^) in the blood of 33 HAT patients and 27 controls (surveys 1 and 2 combined). For each box plot, the features limits correspond, from bottom to top, to the 90^th^, 75^th^, 50^th^ (median), 25^th^ and 10^th^ percentiles.

The general profile of lymphocyte subsets common to both surveys (CD19, CD3, CD4, CD8) is presented in figure 1 for 33 HAT patients and 27 controls. Patients had significantly larger numbers of B (CD19^+^) cells (Table 2) and significantly fewer T (CD3^+^) cells (Figure 1) than controls (*P* = 0.003 and 0.04, respectively). The CD4/CD8 ratios of patients and controls were similar (Table 3). Lymphocyte subsets did not differ significantly between S-1, S-int and S-2 patients (*P*>0.05).

**Table 2:** B cells (CD19^+^) and B-cell activation markers (CD69^+^ and CD95^+^) in peripheral blood mononuclear cells for surveys 1 and 2.

Surface antigen			Blood B cells	
			controls	patients
			n = 27	n = 33
**CD19^+^**	**(1)**		8.7±2.3*	17.2±5.6*
			**n = 14**	**n = 16**
	**(2)**	**CD69^+^**	2.4±1.5*	2.3±3.0*
		**CD95^+^**	4.8±1.8*	11.3±3.8*

(1) n = 33 patients and 27 controls from both surveys.

(2) n = 16 patients and 14 controls from survey 2. Data are expressed in percentages of total events acquired with the FACSCalibur (10^4^ on average)±standard deviation.

* Significant difference between controls and patients (*P*<0.05).

**Table 3:** T cells subsets (CD4^+^/CD8^+^) and T-cell activation markers (HLA-DR^+^, CD69^+^, CD25^+^, CD45RA^+^, CD45RA^−^, CD62L^−^) in peripheral blood mononuclear cells for surveys 1 and 2.

Blood T cells				
Surface antigen			controls	patients
			n = 27	n = 33
**CD4/CD8**	**(1)**		1.1±0.8	1.3±0.6
			**n = 13**	**n = 17**
**CD4^+^**	**(2)**	**HLA-DR^+^**	23.0±4.2	26.9±5.0
**CD8^+^**		**HLA-DR^+^**	45.5±14.8	45.9±12.6
**CD8^+^**		**CD69^+^**	1.2±0.5	1.0±0.4
			**n = 14**	**n = 16**
**CD4^+^**	**(3)**	**CD25^+^**	3.0±1.0*	0.3±0.2*
**CD8^+^**		**CD25^+^**	0.4±0.5	0.2±0.1
			**n = 13**	**n = 17**
**CD4^+^**	**(2)**	**CD45RA^+^**	12.9±6.9	10.3±5.6
**CD4^+^CD62L^+^**		**CD45RA^+^**	19.9±9.0*	30.1±12.8*
**CD8^+^**		**CD45RA^+^**	48.3±21.1	41.9±15.6
**CD8^+^CD62L^+^**		**CD45RA^+^**	12.2±11.6*	23.3±12.3*
**CD4^+^CD62L^+^**		**CD45RA^−^**	24.7±12.2*	42.8±15.7*
**CD8^+^CD62L^+^**		**CD45RA^−^**	15.2±12.3	21.2±11.2
**CD8^+^ CD62L^−^**		**CD45RA^+^**	87.8±11.6*	76.7±12.4*
**CD8^+^ CD62L^−^**		**CD45RA^−^**	84.8±12.4	78.7±11.1

(1) n = 33 patients and 27 controls from both surveys.

(2) n = 16 patients and 14 controls from survey 2.

(3) n = 17 patients and 13 controls from survey 1. Data are expressed in percentages of total lymphocyte events acquired with the FACSCalibur (10^4^ on average)±standard deviation.

* Significant difference between controls and patients (*P*<0.05).

The results for B cells (CD19^+^) in 33 patients and 27 controls (survey 1 and 2) and for B-cell activation markers (CD69^+^, CD95^+^) in 16 patients and 14 controls (survey 2) are presented in Table 2. Patients displayed significantly stronger expression of B-cell activation markers than did controls (*P* = 0.03 and 0.0001, respectively).

The results for CD4^+^ and CD8^+^ T cells (CD3^+^) in 33 patients and 27 controls (surveys 1 and 2) and for T-cell activation markers are presented in Table 3. The results for lymphocyte markers analyzed exclusively in survey 1 (CD25, CD95) or survey 2 (CD45RA, CD69L, CD28, HLA-DR) were analyzed separately. We analyzed the results from 17 patients and 13 controls in survey 1 and 16 patients and 14 controls in survey 2 (Table 3).

NK cells were defined as CD56+ lymphocytes in survey 1 and as CD16+ lymphocytes in survey 2. The relative percentages in patients and controls were therefore compared separately in surveys 1 and 2. No differences were observed between patients and control (*P*>0.05, data not shown).

The percentages of CD4 and CD8 T cells were similar in patients and controls. Similar levels of T lymphocyte activation, as shown by evaluations of CD4^+^HLA-DR^+^, CD8^+^HLA-DR^+^, CD4^+^CD69^+^ and CD8^+^CD25^+^ cells, were observed in patients and controls (Table 3). However, CD4^+^CD25^+^ T-lymphocyte levels were significantly lower in patients than in controls (*P* = 0.02). The proportions of the naive CD4^+^CD45RA^+^ and CD8^+^CD45RA^+^ T-cell subsets were similar in patients and controls, but the proportion of naive T cells expressing CD62L was higher in patients than in controls (*P* = 0.03). Similarly, patients had larger numbers of memory CD4^+^CD45RA^−^CD62L^+^ cells (*P* = 0.01). The pre-effector T lymphocytes populations (CD8^+^CD45RA^−^CD62L^−^) were of similar size in patients and controls (Table 3), but patients had fewer effector T lymphocytes (CD8^+^CD45RA^+^CD62L^−^) (*P* = 0.01).

#### CSF lymphocyte subset profiles and correlation with disease stage

**Figure 2:**
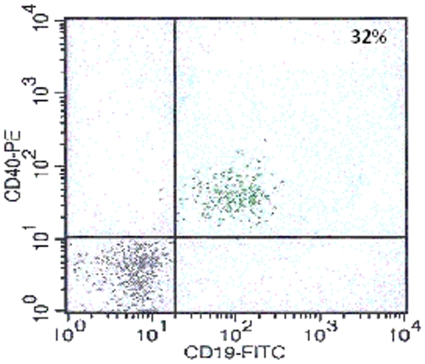
Results of CSF analysis by flow cytometry in a HAT patient with a cell count of 63 cells/µl.

Logistic problems with CSF cell storage before FC analysis resulted in some results being unexploitable. We therefore restricted our analysis to interpretable results. We thus present, in Table 4, only the results for B cells (CD19^+^) for 4 controls, 8 S-1, 8 S-int and 13 S-2 patients. An example of the FC images obtained is provided in Figure 2.

**Table 4:** B cells (CD19**^+^**) in the CSF of HAT patients and controls.

	Control group n = 4	S-1 patients n = 8	S-int patients n = 8	S-2 patients n = 13
**Total CSF cell count ** ***(cells/µl)***	1.7±1.3	2.4±0.9	8.3±1.7	477.7±426.6
**CD19^+^ B-cell count ** ***(cells/µl)***	0.01±0.01*	0.11±0.10¤	0.5±0.36•	243.70±254.26* ¤ •
**CD19^+^ B-cell %**	0.7±0.5*	4±5¤	5.9±5.8•	51.1±23.3* ¤ •

HAT Patients were at stage 1 (S-1), intermediate stage (S-int) or stage 2 (S-2). B cells (CD 19^+^) are expressed as means±standard deviation of absolute values and as a percentage of total CSF cell count. Note: *P*<0.05 for statistical differences.

* S-2 patients *vs* control.

¤ S-1 *vs* S-2 patients.

• S-int *vs* S-2 patients.

No B cells (CD19^+^) were detected in controls (<0.01 cells/µl). The percentages of B cells in CSF samples from controls, S-1 and S-int patients were significantly lower than those in S-2 patients (*P*<0.01). In S-2 patients, B cells accounted for 50% of the cells present (243 cells/µl). The number of B cells increased between control and S-1 (factor of 10), between S-1 and S-int (factor of 5) and between S-int and stage 2 (factor of 500), although the differences between subgroups of patients were not significantly different.

## Discussion

We provide here the first description of differences in lymphocyte subsets in blood and CSF between HAT patients and controls from the same ethnic group and geographic area. Despite the preliminary nature of our results, the small sample size and the logistic problems encountered, we identified several interesting features consistent with known data and experimental findings.

Patients had larger numbers of B cells (CD19^+^) and activated B cells (CD19^+^CD69^+^) than controls, consistent with the immune response classically reported for HAT, with the extensive polyclonal activation of cell likely to proliferate and to secrete large amounts of antibodies [14], [15]. We clearly observed an upregulation of Fas (CD95) expression on the B cells of patients, consistent with the activation of cells via CD95, which may be involved in subsequent lymphocyte apoptosis. Cell activation seems to be self-regulated by the increase in B-cell apoptosis, as described in *T. cruzi* infection [16], [17], suggesting poor conversion of B cells into memory B cells [18]. Radwanska *et al.* (2008) [19] recently demonstrated that the parasite causes a significant decrease in various B-cell populations in mice. These changes may account for the inability of the host to develop protective anti-parasite antibody responses and the lack of development of an effective B-cell memory response against parasites with variant antigens.

This strong B-cell response during infection may affect the homeostasis of T-cell subsets. This phenomenon has been observed in HIV infections [20], [21]. HAT-infected patients have higher percentages of T cells than HIV patients, with no change in CD4/CD8 ratio and an increase in the proportion of naive T (CD62L^+^CD45RA^+^) cells [22]. Indeed, the CD4/CD8 ratio has been shown to increase in autoimmune diseases and to decrease during viral infections, but remains unaffected in HAT. Naive lymphocytes, when stimulated by an antigen, develop into memory lymphocytes, which subsequently differentiate into effector lymphocytes. This process facilitates the rapid and effective secondary response required for long-term protection of the host [23], [24]. We found that the proportion of CD8 T effector cells (CD8^+^CD45RA^+^CD62L^−^) was lower in patients than in controls. Patients also had fewer CD4^+^CD25^+^ cells than controls, accounting for the lower level of activation/proliferation of CD4 T cells in patients. These findings suggest that there may be a T-cell response dysfunction, in patients, possibly due to continuous stimulation of the immune system by new antigenic variants from trypanosomes, resulting in a lack of memory and effector lymphocytes. Thus, the adaptive immune response seems to be impaired in HAT. T cells act as effectors either through cell-mediated cytotoxicity, following the release of dense cytoplasmic granules containing perforin and granzymes, or through the production of cytokines, including IFN-γ in particular, thereby activating other effector cells, such as antigen-presenting cells. On the other hand, some of CD25-expressing T cells belong to the Treg subset populations that represent 10% of the CD4 T cells in humans. The natural Treg cells, CD4^+^CD25^high^Foxp3^+^ were recently studied in experimental model of African trypanosomiasis in *Trypanosoma congolense -* infected mice [25]. This study demonstrated that Treg cells play an important role in trypanotolerance, through a decrease of INF-γ production and downregulation of the inflammatory response depending on IL-10 [25]. Experiment with injection of anti-CD25 on animals before challenge, resulted in the absence of development of parasitemia and elimination of all parasites [26].

Furthermore, the proportion of NK cells was not higher in patients than in controls suggesting that this cell subset of innate immune response was not modified in HAT. However, these results require confirmation with a larger cohort and specific markers.

Finally, the B- and T-cell subset profiles observed are consistent with the classical immunomodulatory mechanisms described in experimental models of HAT [27]–[29]. Direct (parasite-released) and indirect (host cells) mechanisms of immunomodulation are observed. The trypanosome-derived lymphocyte triggering factor (TLTF), also known as trypanin, induces the production of TGF-β mRNA in CD8 T cells and has an immunosuppressive effect [30]. In lymph node T-cell proliferation responses, the classical subset of macrophages plays a central role in inhibiting T-cell proliferation. Early-activated macrophages (by IFN-γ activation, Th1 cytokine pattern) inhibit T-cell proliferation most strongly, through several mechanisms: *i*) the production of prostaglandin, leading to the abolition of IL-2 production [31], *ii*) the induction of a prostaglandin-independent mechanism accounting for the abolition of IL-2 receptor (IL-2R/CD25) expression [32] and *iii*) NO synthesis induced by IFN-γ and resulting in impaired mitogen-induced T-cell proliferation in the spleen, peritoneal cavity and lymph nodes of *T. brucei*-infected mice [33]. Later in the course of infection, T-cell proliferation in lymph nodes may be inhibited through other mechanisms, mediated by an NO-independent pathway and depending on IFN-γ release from CD8 T cells [33], [34]. Alternatively activated macrophages have also been reported to play a role in experimental models of African trypanosomiasis [12], [33]. These cells are activated in the presence of IL-4 and contribute to the development of a Th2 cytokine pattern, which decreases inflammatory responses in the host [35]. Together, these mechanisms impair T-cell activation, resulting in a hyporesponsiveness of T cells to parasite-related and unrelated antigens [12], [33].

We report here the first analysis of the lymphocyte subsets expressed in patients with HAT. The relationship between lymphocyte phenotype and final clinical outcome remains unclear, but our results suggest that various immunosuppressive mechanisms may enable parasites to evade the immune system long enough to cross the blood-brain barrier and hide within the CNS [36]. Logistic problems prevented a more comprehensive analysis of the CSF samples, but our results for this small sample strongly suggest that the progressive increase in the proportion of activated B lymphocytes in the CSF may be an important indicator of stage 2 disease.

Finally, our findings provide new insight into the immunopathological mechanisms underlying HAT and highlight the importance of B lymphocytes for the diagnosis of CNS invasion and disease. B lymphocytes marked with beads coupling with anti-CD19 can be visualized under the microscope for fresh lumbar puncture samples, and this detection could be used in a field-adapted diagnostic tool for determining the stage of the disease. We are currently validating these results in a larger sample.

## Materials and Methods

### Subjects

The study was approved by the Angolan and Gabonese ministries of health and was conducted in accordance with the Helsinki declaration. Informed, written consent was obtained from all included subjects. Subjects were selected actively, during field surveys in Angola and Gabon, and passively, at the reference hospitals at Viana (Angola) and N'kembo (Gabon). Two surveys were carried out in 2002 (survey 1) and 2004 (survey 2). Patients were selected on the basis of whole-blood screening for antibodies with the card agglutination trypanosomiasis test (CATT) [37]. Patients were defined as subjects with a positive CATT test confirmed by parasite detection. Controls were obtained in the same area of endemic disease and had a negative CATT test, with no detection of parasites in the blood after the use of concentration techniques. Subjects presenting evidence of coinfections (malaria, filariasis, HIV, syphilis) or under the age of 10 years were excluded from this study. All subjects were examined by a doctor and clinical examination findings were noted on a case report form.

### Sampling

Blood was obtained during normal diagnostic procedures. We collected 10 ml of blood by venipuncture. The blood sample was then divided in two, with one tube used for subtyping by immunostaining and flow cytometry (FC) (tube containing EDTA) and the other (tube without anticoagulant) used for serological analysis. The storage of blood samples for FC analysis differed between the two surveys:

- In survey 1, staining was carried out with whole blood and monoclonal antibodies (mAbs), as described below, in the field and was then fixed in Cytocheck® (Dako France, Trappes, France) and stored at +4°C until analysis within five days.

- In survey 2, peripheral blood mononuclear cells were isolated in the field by density gradient centrifugation in Vacutainer® CPT™ cell preparation tubes containing sodium heparin (Becton Dickinson, Franklin Lakes, NJ). These cells were then frozen in the presence of fetal bovine serum-10% dimethyl sulfoxide (Sigma-Aldrich, Lyons, France), in liquid nitrogen.

CSF samples were obtained from patients during normal diagnostic procedures. Four CSF samples were obtained from patients consulting for disturbed sleep with minor neurological signs but no detection of trypanosomes in the blood or CSF and a normal CSF cell count. We collected 6 ml of CSF into a conical tube. We centrifuged 4 ml of the sample and dispensed the supernatant into aliquots, which were stored in liquid nitrogen. The remaining 2 ml of the CSF sample was used to determine disease stage in the field. Lumbar puncture samples containing blood (>500 red blood cells/µl) were discarded and the patients from whom such samples were obtained were excluded from the study. CSF samples were immediately stained for FC, fixed with 10% CellFix (Becton Dickinson) and stored at +4°C.

### Parasitological and stage determination procedures

Positive CATT results were confirmed by parasite detection in blood or swollen lymph nodes. A modified miniature anion-exchange centrifugation technique was used for blood [38]. Lymph nodes were punctured and the fluid obtained was observed under the microscope.

Disease stage was diagnosed on the basis of clinical examination and lumbar puncture. CSF was examined immediately after puncture. Cells were counted on Kova slides (Hycor Biomedical Inc., Gardengrove, CA). Parasite detection was carried out by microscopic examination of 2 ml of CSF, after simple centrifugation [39].

(*i*) Stage 1 (S-1) was defined as the presence of fewer than 5 cells/µl in CSF, and the absence of trypanosomes on microscopic examination.

(*ii*) Stage 2 (S-2) was defined as the presence of more than 20 cells/µl CSF and/or the presence of trypanosomes on microscopic examination.

(*iii*) Patients were classified as having intermediate stage disease (S-int) if their CSF profile differed from those described above - *i.e*, between 5 and 20 cells/µl - or if trypanosomes were observed on microscopy but there were fewer than 5 cells/µl CSF [40].

Clinical examination was performed jointly to confirm the diagnosis. The detection of neurological signs in S-int patients was indicative to treat with second stage drugs.

### FC analysis

Samples were analyzed at the CIRMF in Gabon (2000 km from the site of enrollment and sampling in Angola), within five days of sample collection. The mAbs used were obtained from Beckman Coulter (Fullerton, CA, USA) and Becton Dickinson (BD, Franklin Lakes, NJ, USA) (see Table 5 and 6). The results of survey 1 determined the selection of mAbs for use in survey 2.

**Table 5:** Monoclonal antibodies (mAbs) used in surveys 1 and 2. Source: BC, Beckman Coulter (Fullerton, CA, USA); BD Becton Dickinson (Franklin Lakes, NJ, USA).

**Panels survey 1**	**Antibodies**	**Fluorescence**	**Sources**
CD19/CD40/CD3	*J4.119/MAB89/UCHT1*	FITC/PE/PC5	BD/BC/BC
CD19/CD56/CD3	*J4.119/N90/UCHT1*	FITC/PE/PC5	BD/BC/BC
CD4/CD8	*13B8.2/B9.11*	FITC/PC5	BC
CD4/CD25/CD8	*13B8.2/B1.49.9/B9.11*	FITC/PE/PC5	BC
CD3/CD25/CD8	*UCHT1/B1.49.9/B9.11*	FITC/PE/PC5	BC
**Panels survey 2**	**Antibodies**	**Fluorescence**	**Sources**
CD4/CD8	*13B8.2/B9.11*	FITC/PC5	BC
CD3/CD8/CD45RA/CD62L	*UCHT1/SK1/5H9/SK11*	APC/PerCP/FITC/PE	BD
CD3/CD4/CD45RA/CD62L	*UCHT1/L200/5H9/SK11*	APC/PerCP/FITCPE	BD
CD3/CD8/CD45RA/CD28	*UCHT1/SK1/5H9/CD28.2*	APC/PerCP/FITC/PE	BD
CD3/CD4/CD28/HLA DR	*UCHT1/L200/SK1/G46-6*	APC/PerCP/FITC/PE	BD
CD3/CD4/CD28/CD95	*UCHT1/L200/SK1/UB2*	APC/PerCP/FITC/PE	BD
CD8/CD69/CD16	*RPA-T8/L78/3G8*	APC/PerCP/PE	BD/BD/BC
CD19/CD69/CD95	*H1B19/L78/UB2*	APC/PerCP/PE	BD

Fluorescein-isothiocyanate (FITC), phycoerythrin (PE), phycocyanin (PC5) peridinin chlorophyll rotein (PerCP), or allophycocyanin (APC). Negative controls were performed with unrelated murine mAbs (BC and BD).

**Table 6:** Cell lymphocyte populations targeted in surveys 1 and 2.

Target population	Specificity	Survey
CD3^+^	T cells	1 and 2
CD3^+^CD4^+^	Helper T-cell subset	1 and 2
CD19^+^CD69^+^,	Activated B cells	2
CD19^+^CD95^+^	Activated B cells	2
CD3^+^CD4^+^CD25^+^	Activated CD4 T cells	1 and 2
CD4^+^CD62L^+^CD45RA^+^	Naive T CD4 cells	2
CD4^+^CD62L^−^	Pre-effector T CD4 cells	2
CD4^+^HLA-DR^+^	Late-activated T CD4 cells	2
CD3^+^CD8^+^	Cytotoxic T lymphocytes	1 and 2
CD3^+^CD8^+^CD25^+^	Activated cytotoxic T lymphocytes	1 and 2
CD8^+^CD62L^+^CD45RA^+^	Naive CD8 T cells	2
CD8^+^CD28^+^CD45RA^−^	Effector CD8 T cells	2
CD8^+^CD62L^−^	Pre-effector CD8 T cells	2
CD16^+^	Natural killer cells	2
CD3^−^CD56^+^	Natural killer cells	1

Fluorescence intensity was determined with a three-color fluorescence-activated cell sorter machine (FACSCalibur, Becton Dickinson, Franklin Lakes, NJ, USA) in survey 1 and with a four-color machine in survey 2. Lymphocyte cells were gated on the basis of forward and side light-scattering properties and positively selected on the basis of cluster of differentiation (CD) molecule expression. Isotype-matched control mAbs were used to define background fluorescence. Before data acquisition, we checked and optimized the instrument parameters with CaliBRITE Beads (Becton Dickinson). On average, 10^4^ events were acquired with FACSCalibur and analyzed with CELLQUEST software (Becton Dickinson).

During survey 1, we stained 50 µl of total blood from each sample (using 2 µl for each antibody) within 20 minutes. The blood sample was then lysed with a lysis buffer (8.29 g ammonium chloride, 1 g potassium bicarbonate, 0.037 g EDTA in 1l of distilled water, pH adjusted to 7.2), and washed twice with PBS to select leukocytes only. During survey 2, lymphocytes (10^4^) were retrieved from frozen peripheral blood mononuclear cells by thawing and washed in PBS. Their viability was then checked with trypan blue and they were stained with 2 µl mAbs within 20 minutes. All samples were fixed in 4% PFA and analyzed within 5 days. For CSF, we added 2 µl of mAbs to 100 µl of CSF. We then incubated this mixture for 15 minutes at +4°C, fixed the cells and analyzed them within 5 days.

### Statistical analysis

The proportions (% of total events acquired) of each lymphocyte type were calculated after acquisition on a FACSCalibur machine.

The markers used in both surveys (CD19, CD3, CD4, CD8, CD95) were analyzed for all the patients and controls tested, whereas markers used only in survey 1 (CD56, CD25, CD40) or in survey 2 (CD45RA, CD69L, CD28, CD16, HLA-DR) were analyzed separately. Due to the small sample size, the Mann-Whitney non parametric U test was used for comparisons, with P values below 0.05 considered statistically significant.
